# Association between drinking status and risk of kidney stones among United States adults: NHANES 2007–2018

**DOI:** 10.1186/s12889-024-18307-1

**Published:** 2024-03-15

**Authors:** Baian Wei, Wenyue Tan, Shuien He, Shijian Yang, Chiming Gu, Shusheng Wang

**Affiliations:** 1 The Second School of Clinical Medical , Guangzhou University of Chinese Medicine, Guangzhou, 510006 China; 2 The Second Affiliated Hospital of Guangzhou University of Chinese Medicine, Guangdong Provincial Hospital of Chinese Medicine, Guangzhou, 510120 China

**Keywords:** Kidney stones, Drinking status, National health and nutrition examination survey, Alcohol consumption

## Abstract

**Objective:**

This study aimed to investigate the relationship between drinking status and kidney stones occurrence among United States (US) adults who consume alcohol.

**Methods:**

We conducted a cross-sectional analysis using data from the National Health and Nutrition Examination Survey (NHANES 2007–2018). Questionnaires yielded information on alcohol consumption and kidney health. Drinking status was categorized into four groups—former, mild, moderate, and heavy—based on alcohol consumption patterns. The aim was to explore the relationship between drinking status and the prevalence of kidney stones occurrence. For this analysis, we examined a group of individuals diagnosed with kidney stones. With survey weights applied, the total weight of the group was 185,690,415.

**Results:**

We used logistic regression to measure the relationship between drinking status and the likelihood of developing kidney stones. In a fully adjusted model, former drinkers were less likely to have previously experienced kidney stones (OR 0.762, 95% CI 0.595–0.977, *P* < 0.05). In subgroup analysis, heavy alcohol consumption was associated with a significantly reduced likelihood of kidney stones occurrence in various populations. The adjusted odds ratios (with 95% confidence intervals) of kidney stones risk for heavy alcohol consumption were 0.745 (0.566–0.981) for young individuals, 0.566 (0.342–0.939) for older individuals, 0.708 (0.510–0.981) for individuals of white race, 0.468 (0.269–0.817) for individuals with underweight/normal BMI, 0.192 (0.066–0.560) for widowed people, 0.538 (0.343–0.843) for smoking individuals, 0.749 (0.595–0.941) for individuals without a cancer history, and 0.724 (0.566–0.925) for individuals without a stroke history.

**Conclusions:**

In US adults who consume alcohol, a negative linear relationship is apparent between drinking status and the prevalence of kidney stones, with heavy drinking showing a lower prevalence compared to former drinkers. However, the causal relationship between drinking status and kidney stones requires further investigation in future research endeavors.

## Introduction

Kidney stone is a significant health problem in the United States (US), and its prevalence is expected to increase [1, 2]. Its cost amounts to billions of dollars each year [3], and it impairs the quality of life [4]. In some severe cases, it may even be life-threatening [5]. A primary focus of treatment and prevention for kidney stones is increasing fluid intake, particularly water consumption, to promote urine dilution [6]. Nevertheless, the impact of various fluid intake on urine dilution remains ambiguous. Although alcoholic intake is consistently correlated with kidney stones, its relation to the disease remains a matter of controversy. Previous research has indicated that alcohol consumption may decrease the occurrence of kidney stones [7–10]. However, there are inconsistencies in studies regarding the safeguarding effect of consuming alcohol against the condition [11–14]. 

Therefore, with the aim of understanding the potential association between drinking status and kidney stones development better, we conducted a study using the National Health and Nutrition Examination Survey (NHANES) database.

## Materials and methods

### Study population and design

#### Data source and participants

This study utilized data from the NHANES survey conducted by NCHS (National Center for Health Statistics). The NHANES survey received ethical approval from the NCHS Ethics Review Board, and all participants provided informed consent. Six consecutive two-year cycles (2007–2018) of the NHANES survey, which included questions about kidney stones history, were analyzed. Participants enrolled in this study were based on the following participant selection: (1) Participants aged 20 years or older were included. (2) Only individuals with a documented history of at least one prior kidney stone occurrence were considered for the analysis. (3) Participants with missing alcohol intake data, missing diet data, and those who did not answer the kidney stones question were excluded. (4) Participants with a lifetime alcohol intake of fewer than 12 drinks were excluded.

### Variable

#### Drinking status

Alcohol intake status was delineated into four distinct categories: former drinkers, indicating individuals who abstained from alcohol consumption within the past year but had consumed a minimum of 12 drinks throughout their lifetime; mild drinkers, characterized by an average intake of no more than 1 drink per day for women and 2 drinks per day for men during the preceding year; moderate drinkers, denoting individuals with an average intake of no more than 3 drinks per day for women and 4 drinks per day for men in the past year; and heavy drinkers, representing those who consumed an average of 4 or more drinks per day for women and 5 or more drinks per day for men throughout the previous year.

#### Kidney stones

The study’s outcome was defined as whether participants had ever experienced kidney stones. This was evaluated by asking the question, “Have you ever had a kidney stone?”, with those who answered “yes” considered to have a history of kidney stones.

#### Dietary intake

The present study assessed the types and quantities of all foods and beverages consumed by NHANES participants during the 24 h preceding their interview, from midnight to midnight. Dietary intake data obtained from the first day of 24-hour dietary recall interviews was used to estimate the intake of energy, nutrients, and other food components. The selected independent variables for this study were Energy, Protein, Carbohydrate, Vitamin C, Vitamin D, Vitamin E, Calcium, and Magnesium. More data is referred to the official website of NHANES.

#### Other definitions

Information on age, sex (Male, Female), race-ethnicity (White, Black, Mexican American, Other Race), education levels (Less Than 9th Grade, 9-11th Grade (includes 12th grade with no diploma), High School Graduate/Ged or Equivalent, Some College or AA Degree, College Graduate or Above), smoking status (No, Yes) was obtained through self-report from study participants. Additionally, participants were asked to report any prior doctor diagnoses of heart attack, stroke, or cancer. In this study, diabetes was defined based on several factors: a doctor’s diagnosis of diabetes, glycohemoglobin HbA1c level greater than 6.5%, fasting glucose level greater than or equal to 7.0 mmol/L, random blood glucose level greater than or equal to 11.1 mmol/L, or two-hour OGTT blood glucose level greater than or equal to 11.1 mmol/L. Participants with diabetes were further subdivided into three groups based on these criteria: those with diabetes mellitus (DM), those with impaired fasting glucose (IFG), and those with impaired glucose tolerance (IGT). Body mass index (BMI) was calculated as weight (in kilograms) per squared height (in meters). Finally, the ratio of income to poverty (RIP) was calculated based on family income.

### Statistical analysis

A statistical analysis was conducted in accordance with guidelines from the Centers for Disease Control and Prevention (CDC) (NHANES Tutorials (cdc.gov)) [15]. NHANES dietary subsample weights were used in statistical analysis. Sample weight was applied to each participant in the analysis [16]. We examined associations between kidney stones and other variables using weighted logistic regression. Descriptive analyses were conducted for all participants, reporting means (SE) for continuous variables and percentages (SE) for categorical variables. Categorical variables were analyzed using logistic regression with confidence intervals computed on the log-odds scale. Continuous variables were analyzed using linear regression, estimating 95% confidence intervals based on standard error of the mean.

Weighted stratified logistic regression models were used to performe subgroup analysis and Interaction terms were used between subgroup indicators to test the effect modification in subgroups. The stratification factors included age (20–59, ≥ 60), BMI (underweight/normal:<25 kg/m^2^, overweight:25-30 kg/m^2^, obesity:≥30 kg/m^2^), race, marriage status, smoking status, heart attack, cancer, stroke, diabetes. Statistical analysis was conducted using R 4.1.2 and Free Statistics software version 1.9. A two-tailed test was performed and *p* < 0. 05 was considered statistically significant.

## Result

### Baseline characteristics of participants

**Fig. 1:**
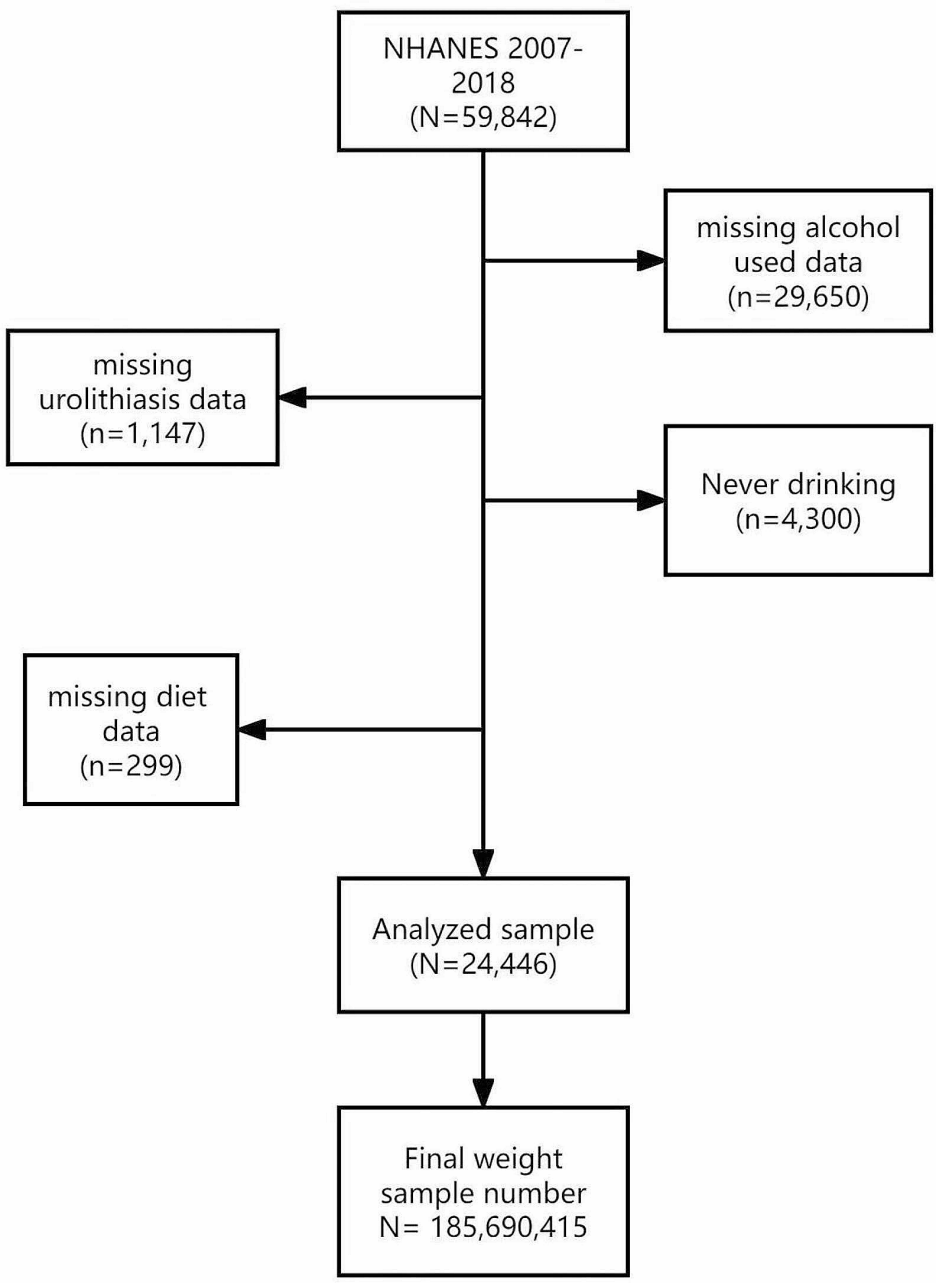
The participant flow chart

The analysis included 24,446 participants (total weighted *n* = 185,690,415) aged 20 years or older, who had previously passed at least one kidney stone. Ultimately, 29,650 participants with missing alcohol intake data, 4,300 with a lifetime alcohol intake of fewer than 12 drinks, 299 missing diet data, and 1,147 individuals younger than 20 who did not answer the kidney stone question were excluded from analysis. Sample weights were calculated to account for complex survey design, including oversampling, survey nonresponse, and post-stratification effects to ensure accurate representation of the U.S. civilian noninstitutionalized population (Fig. 1).

Table 1 presents a cross-sectional characterization of 23,927 participants categorized based on alcohol consumption patterns, including former drinking, mild drinking, moderate drinking, heavy drinking, and the total population. Various demographic, health, and nutritional parameters underwent statistical comparisons among drinking strata, with p-values indicating the significance of differences between strata.

All factors demonstrated significant disparities across drinking statuses (*P* < 0.05). For instance, compared to former drinkers, heavy drinkers exhibited trends towards younger age, male gender, unmarried status, and higher body mass indices. Moreover, increased consumption of total energy, protein, carbohydrates, and calcium was observed in the heavy drinking groups. Disease epidemiology also exhibited considerable variations, with elevated rates of diabetes, myocardial infarction, and nephrolithiasis observed among former drinkers in comparison to other categories.

**Table 1:** The population characteristics

		Drinking status				
Variable	Total	Former	Mild	Moderate	Heavy	P
**Age(years)**	47.07(0.26)	55.43(0.38)	50.78(0.32)	44.23(0.40)	38.29(0.34)	< 0.001
Age Group(years)						< 0.001
20–59	16,243(74.90)	2108(11.05)	5772(36.70)	3402(21.88)	4961(30.37)	
≥60	7680(25.10)	2349(24.34)	3683(54.45)	933(13.66)	715(7.55)	
**Sex**						< 0.001
Female	11,290(49.19)	2181(15.27)	4068(37.52)	2714(25.98)	2327(21.23)	
Male	12,633(50.81)	2276(13.52)	5387(44.68)	1621(13.85)	3349(27.94)	
**Race**						< 0.001
White	10,617(68.84)	1995(14.17)	4455(43.09)	1875(20.16)	2292(22.57)	
Black	5087(10.68)	1021(15.92)	2035(40.60)	1005(21.11)	1026(22.36)	
Mexican	3498( 8.21)	672(14.89)	927(24.64)	647(19.20)	1252(41.27)	
Other Race	4721(12.27)	769(13.90)	2038(41.81)	808(17.18)	1106(27.11)	
**Marriage status**						< 0.001
Married	12,090(53.67)	2376(15.53)	5492(47.66)	2050(19.28)	2172(17.54)	
Never Married	4499(19.27)	451( 7.82)	1429(31.32)	952(19.97)	1667(40.89)	
Widowed	1604( 5.07)	578(29.57)	655(42.93)	210(17.26)	161(10.24)	
Living With Partner	2111( 8.66)	284(10.45)	598(27.83)	433(22.80)	796(38.92)	
Divorced	2794(10.88)	589(15.82)	1029(38.92)	555(21.49)	621(23.77)	
Separated	815( 2.42)	177(17.12)	248(29.56)	132(17.22)	258(36.10)	
**Education levels**						< 0.001
Less Than 9th Grade	1966( 3.91)	689(34.39)	509(24.11)	250(12.50)	518(28.99)	
9-11th Grade (Includes 12th Grade With No Diploma)	3303( 9.94)	877(24.16)	887(28.46)	480(14.64)	1059(32.74)	
High School Graduate/Ged or Equivalent	5480(22.79)	1130(17.35)	1882(35.02)	947(18.63)	1521(29.00)	
Some College or AA Degree	7375(32.63)	1141(12.69)	2956(39.00)	1479(20.12)	1799(28.20)	
College Graduate or Above	5783(30.70)	614( 8.26)	3216(54.29)	1178(22.99)	775(14.46)	
**RIP**						< 0.001
<1	4384(12.81)	1038(19.33)	1204(27.74)	695(16.27)	1447(36.67)	
1–5	13,414(54.33)	2649(16.27)	5240(39.40)	2419(19.13)	3106(25.20)	
≥5	4187(26.35)	383( 7.67)	2240(50.99)	900(23.62)	664(17.72)	
BMI(kg/m^2)^	29.11(0.10)	30.35(0.19)	28.89(0.13)	28.67(0.14)	29.10(0.13)	< 0.001
BMI Group(kg/m^2)^						< 0.001
Underweight/Normal	6692(29.17)	1021(11.07)	2742(41.28)	1301(22.41)	1628(25.24)	
Overweight	7845(32.60)	1399(13.74)	3222(43.42)	1373(19.12)	1851(23.72)	
Obesity	9198(37.65)	1960(17.18)	3427(39.12)	1638(18.55)	2173(25.15)	
Disease history						
**Cancer**						< 0.001
No	21,552(89.49)	3843(13.74)	8275(39.92)	4019(20.26)	5415(26.08)	
Yes	2355(10.46)	612(19.94)	1171(51.61)	315(16.10)	257(12.36)	
**Stroke**						< 0.001
No	22,963(97.02)	4064(13.73)	9116(41.24)	4227(20.04)	5556(24.99)	
Yes	932( 2.88)	381(35.47)	332(38.79)	102(12.04)	117(13.70)	
**Diabetes mellitus**						< 0.001
No	17,365(77.68)	2637(12.12)	6851(40.51)	3420(21.00)	4457(26.37)	
IFG	1130( 4.62)	191(12.47)	471(44.79)	186(18.90)	282(23.85)	
IGT	847( 3.16)	218(21.57)	324(42.54)	120(13.55)	185(22.34)	
DM	4340(13.55)	1357(26.02)	1738(43.79)	556(14.55)	689(15.64)	
**Heart attack**						< 0.001
No	22,819(96.51)	4050(13.77)	9004(41.04)	4235(20.17)	5530(25.01)	
Yes	1079( 3.42)	400(31.46)	446(44.88)	98(10.06)	135(13.60)	
**Kidney Stone**						< 0.001
No	21,634(90.34)	3903(13.83)	8467(40.70)	3995(20.11)	5269(25.35)	
Yes	2289( 9.66)	554(19.53)	988(45.38)	340(17.04)	407(18.05)	
**Smoking status**						< 0.001
Never	11,986(51.59)	1961(12.14)	5490(48.52)	2266(19.97)	2269(19.37)	
Former	6417(26.80)	1611(19.04)	2591(41.55)	1099(20.73)	1116(18.68)	
Now	5506(21.57)	882(13.98)	1369(23.06)	966(18.25)	2289(44.71)	
**Diet factors**						
Energy(kcal)	2187.37(8.89)	1997.66(19.89)	2145.61(13.88)	2104.58(18.75)	2434.40(19.66)	< 0.001
Protein(gm)	84.37(0.46)	77.38(1.02)	83.93(0.63)	81.87(0.91)	91.21(1.00)	< 0.001
Carbohydrate(gm)	256.52(1.11)	252.89(2.66)	254.60(1.82)	240.55(2.45)	274.70(2.77)	< 0.001
Vitamin C(mg)	81.97(1.26)	77.97(2.03)	88.25(1.73)	80.02(1.93)	75.37(2.16)	< 0.001
Vitamin D(mcg)	4.65(0.06)	4.84(0.14)	4.77(0.09)	4.37(0.13)	4.57(0.11)	0.01
Vitamin E(mg)	9.01(0.09)	8.10(0.14)	9.46(0.13)	8.81(0.16)	8.96(0.15)	< 0.001
Ca(mg)	982.99(7.07)	942.03(15.98)	976.82( 9.49)	959.28(14.62)	1036.28(14.41)	< 0.01
Mg(mg)	311.42(2.18)	282.81(3.79)	319.65(2.87)	301.55(3.73)	322.33(3.17)	< 0.001

Abbreviation: RIP: the ratio of income to poverty; BMI: body mass index; DM: diabetes mellitus; IFG:impaired fasting glucose; IGT: impaired glucose tolerance

### Drinking status and kidney stones

Table 2 delineates the association between drinking status and kidney stone risk across several regression models with stepwise adjustment for confounding variables. In the unadjusted analysis (Model 1), mild drinkers showed 21% lower odds (OR 0.79; 95% CI, 0.68–0.92), moderate drinkers 40% lower odds (OR 0.60; 95% CI, 0.49–0.73), and heavy drinkers 50% lower odds (OR 0.50; 95% CI, 0.41–0.62) of developing stones compared to former drinkers (all *P* < 0.01). After adjusting for sociodemographics (Model 2) including sex, age, race, marital status, income, and education attenuated some of these associations, with only heavy drinkers retaining 29% lower odds (OR 0.71, *P* < 0.01). Further models adjusted for health factors (Model 3), including stroke, cancer, heart disease, diabetes, and smoking; and nutritional variables (Model 4) of protein, calcium, vitamin C, vitamin D, carbohydrates and others. After fully adjusting for all covariates, heavy drinking remained associated with a 24% reduction in nephrolithiasis odds (OR 0.76; CI: 0.60–0.98; *P* = 0.03), while mild and moderate intakes no longer reached significance.

**Table 2:** The association between drinking status and the risk of kidney stones

	Model 1		Model 2		Model 3		Model 4	
Drinking status	OR (95%CI)	P	OR (95%CI)	P	OR (95%CI)	P	OR (95%CI)	P
Former	ref		ref		ref		ref	
Mild	0.79(0.68,0.92)	< 0.01	0.87(0.75,1.01)	0.07	0.93(0.80,1.09)	0.37	0.95(0.81,1.11)	0.48
Moderate	0.60(0.49,0.73)	< 0.01	0.81(0.65,1.01)	0.06	0.87(0.70,1.08)	0.19	0.89(0.72,1.11)	0.31
Heavy	0.50(0.41,0.62)	< 0.01	0.71(0.56,0.88)	< 0.01	0.73(0.58,0.94)	0.01	0.76(0.60,0.98)	0.03
p for trend		< 0.01		< 0.01		0.01		0.03

**Model 1**: Crude model

**Model 2**: Adjusting for sociolect-demographic variables (sex, age, race, marriage status, RIP, education levels)

**Model 3**: Adjusting for personal status variables (sex, age, race, marriage status, RIP, education levels, BMI, cancer, stroke, diabetes, heart attack, smoke status)

**Model 4**: Fully-adjusted mode, which adjusts for sex, age, race, marriage status, RIP, education levels, BMI, cancer, stroke, diabetes, heart attack, smoking status, energy, protein, carbohydrate, vitamin c, vitamin d, vitamin e, calcium, magnesium

### Results of subgroup analysis

**Fig. 2:**
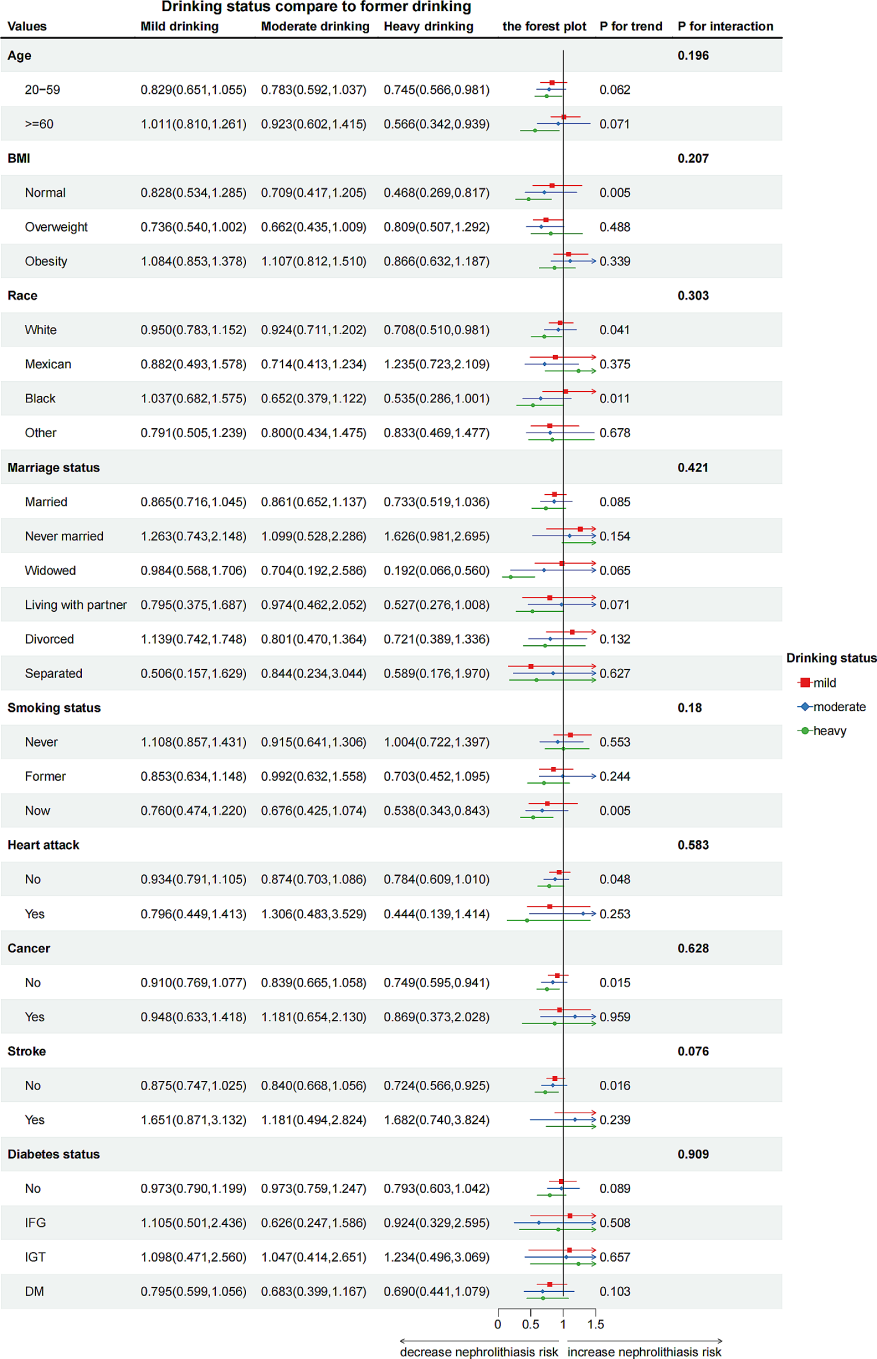
The subgroup analysis. Abbreviation: *RIP*: the ratio of income to poverty: *BMl*: body mass index; *M*: diabetes mellitus; *IFG*: impaired fasting glucose; *IGT*: impaired glucose tolerance

The study performed subgroup analyses to examine the relationship between alcohol consumption and kidney stones prevalence based on confounders. Heavy drinkers had similar kidney stones occurrence in individuals < 60 years (OR = 0.745; 95% CI: 0.566–0.981; *P* = 0.036) and ≥ 60 years (OR = 0.566; 95% CI: 0.342–0.939; *P* = 0.028) compared to moderate drinkers (Fig. 2). A significant disparity was observed between underweight/normal weight individuals heavily consuming alcohol versus previous drinkers (OR = 0.468; 95% CI: 0.269–0.817; *P* = 0.008).

Statistically significant results indicated a positive correlation between heavy-drinking White Americans (OR = 0.708, 95% CI: 0.510–0.981, *P* = 0.039) and smokers (OR = 0.538, 95% CI: 0.343–0.843, *P* = 0.007) versus non/less drinkers. A similar relation was observed in widowed individuals (OR = 0.192, 95% CI: 0.066–0.560, *P* = 0.003). Significant ORs were only present in individuals without cancer (OR = 0.749, 95% CI: 0.595,0.941, *P* = 0.014) or stroke (OR = 0.724, 95% CI: 0.566,0.925, *P* = 0.001). No associations were observed between drinking and kidney stones in individuals with diabetes or heart attack (all *P* > 0.05). No significant interaction effects occurred after adjusting for covariates (all P for interaction > 0.05).

## Discussion

We analyzed the relationship between drinking status and the risk of developing kidney stones using large population data from NHANES. Our findings demonstrate a negative correlation between drinking status and kidney stone risk, with higher drinking status associated with a lower prevalence of kidney stones. Additionally, drinking status indicates a trend toward a decreased risk of kidney stones.

Previous studies have reported a negative association between alcohol consumption and kidney stones-the same finding as ours. According to data from the Oxford cohort of the European Prospective Investigation into Cancer and Nutrition (EPIC), individuals who consumed ≥ 16 g of alcohol per day had a reduced risk of kidney stones compared to those who consumed 1–7 g of alcohol per day (HR = 0.65, 95% CI: 0.47–0.91) [17]. In addition, a study based on UK Biobank data demonstrated a linear decrease in kidney stone risk with increasing alcohol consumption (P for trend < 0.001); the HR (95%CI) per 200 mL/d alcohol was 0.85 (0.82–0.88) [10]. Two recent large cohort studies conducted in China [18] and Korea [8] have also suggested a negative correlation between alcohol consumption and kidney stones risk. Despite previous works demonstrating the relationship between alcohol consumption and kidney stones, the study populations were limited and considered only a few potential confounders [9, 19]. A study based on three ongoing cohorts (HPFS, NHSI, and NHSII) reported that the reduced risk of kidney stones was observed only for the consumption of > 1 serving of beer and wine per day compared with drinking < 1 serving per week, but not for liquor consumption [20]. However, findings from one Guangzhou hospital revealed no significant differences in alcohol consumption between never drinkers, occasional drinkers, and regular drinkers, as well as beer, wine, and hard liquor consumption [21]. Similarly, some researchers reported no significant relationship between alcohol consumption and kidney stones [11–14]. The disparity in findings could be attributed to the various types and levels of alcohol exposure in different nations, as well as the ethnic diversity in those countries. Hence, our findings are more specific to the American population and underscore the linear relationship between drinking status and kidney stones.

Whether alcohol consumption is beneficial or harmful for stone formation is currently unclear. Low urine output is the primary risk factor for stone formation, and this is widely accepted [22]. The observed inverse correlation between drinking status and kidney stone risk could be explained by the diuretic effect of alcohol [23]. Alcohol has been suggested to dilute metabolites in the blood and urine [9, 24], inhibit vasopressin secretion, and ultimately prevent stone formation [25]. Moreover, research from the UK Biobank indicates that a higher intake of fluids, including tea, coffee, and alcohol (but not water), is associated with a decreased risk of urolithiasis [10]. Studies have shown that Randall’s plaque, which is made up of calcium phosphate crystals combined with an organic matrix of proteins and lipids and is present on the kidney papillary surfaces, is associated with the development of calcium oxalate (CaOx) stone. Moderate alcohol intake has been shown to raise circulating levels of high-density lipoprotein cholesterol, apolipoprotein AI and adiponectin, while lowering fibrinogen levels [26]. Moreover, the tissue surrounding Randall’s plaques within the kidney is believed to be associated with the presence of pro-inflammatory macrophages and the downregulation of anti-inflammatory macrophages [27]. What’s interesting is that some researchers point out that moderate alcohol consumption has anti-inflammatory properties that may help reduce the prevalence of urolithiasis to a certain extent [28]. These findings offer some biological evidence to support our findings.

Although our research indicates a negative correlation between drinking status and kidney stones, excessive alcohol intake can still be harmful [29]. Excessive drinking is associated with various adverse health outcomes and can adversely affect health throughout a person’s lifetime [30]. In conclusion, the correlation between alcohol and kidney stones is multifaceted and incompletely understood. Although excessive drinking can elevate the risk of developing other health conditions, moderate alcohol consumption may provide protective effects. Our study revealed decreased kidney stone risk with increased drinking status in adults, demonstrating that intervening in the latter may be beneficial in preventing the former. The findings offer new clues about the potential impact of diet on kidney stone risk. To identify the underlying mechanisms and develop effective strategies to prevent kidney stones, more study is required.

This study has several strengths, including our study had a large sample size of 24,446 participants (total weighted *n* = 185,690,415) aged 20 years or older, ensuring that our findings are representative of the general American adult population. Moreover, to our knowledge, this is the first study to use large-scale public database (NHANES) to study the relationship between drinking status and kidney stones in US adults who consume alcohol. In addition, we employed several statistical modes to comprehensively explore the relationship between drinking status and kidney stone risk from various perspectives, which enhanced the robustness and stability of our results. However, the study we conducted also has some limitations. First, as with prior cross-sectional research utilizing NHANES [31, 32], we were unable to draw causal inferences, and the results were susceptible to selection and response bias. Second, self-reported data can be associated with several biases, including recall bias. Moreover, the information recalled is not always accurate since many cases of asymptomatic stones are excluded, and ureteric and bladder stones are not included. Another limitation was that the type of kidney stones was not identified; therefore, we could not conduct further analyses investigating the various causes of kidney stones and their contribution to the increase. Furthermore, we were unable to analyze common essential risk factors, such as family history and sun exposure levels, which may have influenced our results. Additionally, there could be interactions among drinking status and BMI, race, marital status, and diabetes with respect to kidney stones disease. Nonetheless, our data offered a critical insight into the epidemiology of kidney stones and the underlying factors.

## Conclusion

A negative linear relationship exists between drinking status and the prevalence of kidney stones, and heavy drinking is associated with a lower prevalence of kidney stones compared to former drinkers. These results offer important information about the connection between drinking status and the prevalence of kidney stones and may aid in the identification of new preventative and treatment strategies. Despite these results, a causal relationship between drinking status and kidney stones remains to be studied in future research.

## Data Availability

All data can be obtained from the online NHANES website: https://www.cdc.gov/nchs/nhanes/index.htm.
